# A Narrative Review of Advancements in Magnetic Resonance Imaging (MRI) Technology: Evaluating the Shift From Helium-Cooled to Helium-Free Systems

**DOI:** 10.7759/cureus.96615

**Published:** 2025-11-11

**Authors:** Meshaal M Alenezi, Sultan Alqahtani, Haifa Alqahtani, Sadeem Alasmari, Nora Alyami, Faleh Alenzi, Salem F Alanazi

**Affiliations:** 1 Radiology, King Fahad Hospital-Jeddah (KFHJ), Jeddah, SAU; 2 Nuclear Medicine, King Khalid Hospital (KKHH), Hail, SAU; 3 Medical Ultrasound, Imperial College London, London, SAU; 4 Radiological Sciences, King Saud Bin Abdulaziz University for Health Sciences, Al Ahsa, SAU; 5 Radiology, Al Shefa Specialized Hospital, Najran, SAU; 6 Radiology and Medical Imaging, Prince Sattam Bin Abdulaziz University, Al-Kharj, SAU; 7 Medical Imaging, Northern Area Armed Forces Hospital, King Khalid Military City, SAU; 8 Radiology, Medical Cities Program, General Directorate of Medical Services, Ministry of Interior, Riyadh, SAU

**Keywords:** cryogen-free, helium-free system, magnetic resonance imaging, mid field mr, superconducting magnet

## Abstract

Magnetic resonance imaging (MRI) traditionally relies on large liquid-helium inventories to maintain superconductivity, creating siting complexity and exposing providers to helium-supply volatility. Sealed-helium (zero-boil-off) designs reduce helium volumes to single-digit liters and may eliminate vent-pipe requirements, potentially broadening siting options and lowering operational risk. We conducted a narrative search of major databases and relevant gray literature to summarize the clinical performance, installation logistics, and operational metrics of sealed-helium systems across low, mid, and conventional field strengths. Peer-reviewed clinical studies remain small and heterogeneous. Early reader-blinded analyses suggest diagnostic adequacy for selected protocols at low and mid field, but robust equivalence to 1.5 T/3 T has not been demonstrated. Vendor materials' reports describe conduction-cooled magnets with homogeneity and stability compatible with clinical imaging. However, independent multicenter confirmation is limited. Vendor materials report substantial helium savings and vent-pipe-free installations, which require external validation. Overall, sealed-helium MRI appears operationally attractive and may support expanded access, particularly where siting barriers and helium logistics constrain capacity. To inform clinical and purchasing decisions, multicenter comparative studies and standardized operational benchmarks (energy use, uptime, and unplanned service) are needed alongside transparent reporting of vendor involvement.

## Introduction and background

Over the last two decades, magnetic resonance imaging (MRI) hardware and reconstruction advances have broadened clinical capabilities while reducing acquisition time and improving workflow [1-5]. Conventional superconducting magnets depend on large volumes of liquid helium and vent-pipe infrastructure, which constrain siting, particularly upper-floor and outpatient departments, which also expose providers to supply shortages and refilling logistics. Sealed-helium (zero-boil-off) designs reduce helium inventory to single-digit liters and integrate cryocoolers to maintain superconductivity without routine refill [6-12]. Multiple vendors have introduced sealed-helium systems at low and mid field strengths and have publicized reduced installation complexity and helium independence [13-17].

Despite growing interest, questions remain about clinical diagnostic adequacy compared with standard 1.5 T or 3 T scanners and about the real-world operational profile, including installation requirements, energy consumption, uptime, and service reliability. Peer-reviewed evidence is emerging, but fragmented and heterogeneous, while vendor claims often precede independent validation.

Contemporary MR-suite planning and safety guidance underscores the importance of room zoning, access control, and ferromagnetic safety screening when considering new installations [18]. Recent reviews summarize how diagnostic trade-offs vary by field strength, including 0.55 T, 1.5 T, and 3 T platforms, and outline technical advances that shape performance at lower fields [19,20].

## Review

Objective

This narrative review summarizes peer-reviewed and vendor-reported evidence on sealed-helium MRI across clinical performance and operational domains (installation, helium logistics, energy, and uptime), explicitly separating independently verified data from manufacturer claims. We conducted narrative research, rather than a formal systematic review, to map the evidence base, characterize heterogeneity, and identify gaps that prevent effect estimation at present.

Methods

We performed a narrative review of peer-reviewed and gray literature on sealed-helium MRI from January 1, 2000, to October 8, 2025, across MEDLINE (via PubMed), Embase, Scopus, IEEE Xplore, and targeted manufacturer resources. The search combined controlled vocabulary and keywords for sealed-helium and cryogen-free MRI, low and mid field MRI, and superconducting magnet design. Eligibility focused on the following: (i) clinical performance studies of sealed-helium systems and field strength, (ii) reports of installation and operational outcomes (including helium and inventory, vent-pipe requirements, scanner mass, energy use, and unplanned services), and (iii) engineering bench reports relevant to clinical feasibility (e.g., field homogeneity and stability).

**Figure 1:**
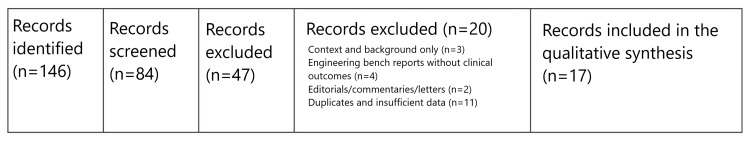
Simplified identification and screening flow for this narrative review A total of 84 records were screened; 47 full texts were assessed. After eligibility, 37 records remained, of which 20 were excluded at the synthesis stage (context and background only; engineering bench reports without clinical outcomes; editorials/commentaries/letters; duplicates and insufficient data), yielding 17 peer-reviewed studies included in the qualitative synthesis.

Two reviewers independently screened records at the title and abstract levels and then at the full-text stage. Disagreements were solved by consensus with a third reviewer. Data were charted using a standardized form covering study design, system characteristics, outcomes, and vendor involvement/funding where reported. Given heterogeneity and early mixed evidence base, we mapped findings by domain without formal risk of bias grading, and we explicitly distinguished vendor-reported claims from independently verified data. Due to limited and heterogeneous clinical studies, a meta-analysis was not performed. The identification and screening approach used for this narrative review is summarized in Figure 1. The flow diagram in Figure 1 summarizes identification and screening. Seventeen peer-reviewed studies met the inclusion criteria and were included in the synthesis. Other records used to provide contextual or vendor information are cited separately in the text but were not included in the count of analyzed studies.

Results

Evidence Identification and Overview

Figure 1 summarizes the record identification and screening steps for this narrative review. The search yielded a small body of peer-reviewed clinical studies, primarily at low and mid field alongside engineering and bench reports describing helium-free magnet design and performance [1-12,14-17]. Besides vendor materials detailing product features, siting specifications, and installation claims [13-17], peer-reviewed reports were reviewed for clinical and operational insights [13-17]. Given the variability in design, indications, and reporting, we summarize the evidence in three streams: (i) clinical performance, (ii) Installation and operation, and (iii) engineering and bench reports.

Clinical Performance

Small cohorts with the reader-blinded image assessments suggest diagnostic adequacy for selected protocols at low and mid field [21-23]. Although minor differences exist in contrast-to-noise ratio and sequence optimization requirements compared to 1.5 T and 3 T scanners, image quality remains clinically acceptable. Indications were limited, for instance, neuro- and musculoskeletal systems, and studies were underpowered to demonstrate formal non-inferiority. Reporting of standardized outcomes, for instance, lesion detectability, reader agreement, and standardized acquisition times, was inconsistent, preventing effect estimation.

Installation and Operational Metrics

Independent site-level reports variably describe single-digit liter helium inventories, the potential elimination of vent-pipe requirements, and a reduction in scanner mass and decrease the installation complexity, which could facilitate upper-floor or outpatient deployment. However, energy use under clinical duty cycles, uptime, and unplanned service events are rarely reported in peer-reviewed sources. Much of this information appears in vendor material and requires independent validation.

Engineering and Bench Reports

Helium-free low-temperature superconducting (LTS) and high-temperature superconducting (HTS) magnets using conduction cooling have demonstrated field homogeneity compatible with clinical imaging over clinically relevant diameters of spherical volume (DSVs), with reported ppm measurements at 1.5-3 T in a bench setting [6-10]. Translation to consistent clinical performance depends on system integration (gradient performance, radiofrequency (RF) chain, sequence optimization) and site conditions.

Table 1 presents the operational differences between conventional and sealed-helium MRI.

**Table 1:** Operational metrics: conventional MRI vs. sealed-helium MRI MRI: magnetic resonance imaging; RF: radiofrequency; OPEX: operational expenditure Source: Author-generated comparative synthesis of operational attributes, derived from manufacturer technical documentation [15-17] (Siemens Healthineers, GE HealthCare, Philips Healthcare) and peer-reviewed engineering sources [9,10]. No new measurements were performed by the authors.

Attribute	Conventional MRI	Sealed‑helium MRI	Operational impact	Notes
Helium volume	Hundreds to 1500 L	≈1-7 L sealed	Eliminates refills	Manufacturer‑reported
Quench pipe	Typically required	Often not required	Simplifies siting/build	Local code-dependent
System mass/siting	Higher; floor reinforcement common	Lower; broader siting	Upper floors/outpatient/mobile	RF shielding still required
Cooling and energy	Higher facility cooling demand	Cryocoolers; optimized duty cycle	Potential OPEX reduction	Energy benchmarks vary
Service/downtime	Quench/refill events possible	Reduced quench logistics	Potential uptime gains	Design/maintenance-dependent

Table 2 lists the engineering characteristics from selected reports.

**Table 2:** Engineering characteristics of cryogen-free MRI magnets MRI: magnetic resonance imaging; DSV: diameter of spherical volume; HTS: high-temperature superconductor; LTS: low-temperature superconductor; NbTi: niobium-titanium; YBCO: yttrium barium copper oxide; Bi-2223: bismuth strontium calcium copper oxide; GM: Gifford-McMahon cryocooler Source: All data derived from peer-reviewed engineering studies [10,11,24-27]. No new measurements were performed by the authors.

Reference	Field strength (T)	Average field decay/drift	Magnet homogeneity (unit; DSV)	Number of coils/configuration	Conductor/wire	Cooling
Shen et al. 2001 [24]	9.5 T	Not reported	<0.1% over 10 mm DSV	5 coils	NbTi (LTS)	Cryocooler (conduction cooled)
Wang et al. 2023 [10]	1.5 T	Not reported	≤12.1 ppm over 450 mm DSV	-	NbTi (LTS)	Conduction cooled
Liu et al. 2025 [25]	3 T	Not reported	4.18 ppm and 1.02 ppm over 180 mm DSV	-	Not reported	GM refrigerator (conduction cooled)
Li and Roell 2021 [26]	14 T	Not reported	1.5 ppm over 400 mm DSV	Not reported	Bi-2223 (HTS)	Conduction cooled
Parkinson et al. 2013 [27]	1.5 T	Not reported	±20 ppm over 120 mm DSV	16 double-pancake coils	YBCO (HTS)	Conduction cooled

Table 3 provides a comparative evidence summary using the A/B/C rubric.

**Table 3:** Comparison of helium-free systems vs. conventional systems using the A/B/C evidence rubric A: highly supportive; B: favorable signal; C: limited or mixed evidence. "Favorable signal" indicates multiple independent reports trending positive but insufficient for firm conclusions. MRI: magnetic resonance imaging Source: Author-generated synthesis; not reproduced from a prior publication.

	Helium-free MRI	Traditional MRI
Cost-effectiveness	B	-
Installation requirements	A	-
Technical parameters and metrics	C	C
Environment and sustainability	B	-

Discussion

Principal Findings

This review maps a small but growing evidence base on sealed-helium MRI. Early clinical studies, largely low and mid field and limited in scope, suggest that diagnostic adequacy can be achieved for selected protocols when sequences are adapted. Formal non-inferiority to 1.5 T and 3 T has not yet been demonstrated across indications. Engineering reports show that conduction-cooled magnets can reach field homogeneity compatibility with clinical imaging, but multicenter confirmation remains sparse. Vendor materials describe clear siting and helium logistics advantages. These claims require external verification.

Clinical Implications

Hospitals and imaging centers looking to expand access, especially outpatient centers or upper-floor sites, may find sealed-helium systems beneficial, as they lower siting barriers by removing routine refills and, in some settings, eliminating the need for vent-pipe requirements. The lower field strengths demand careful sequence selection, attention to contrast-to-noise ratio, and protocol standardization. In facilities where follow-up imaging or motion-tolerant studies dominate the case mix, sealed-helium systems may integrate effectively. However, in centers focused on ultra-high-resolution or susceptibility-sensitive imaging, conventional 1.5 T and 3 T scanners will likely remain the standard [21].

Operational and Sustainability Considerations

The operational rationale is straightforward. Single-digit liter helium inventories reduce exposure to supply shortages and refilling logistics [22]. Potential reduction in magnet mass and infrastructure can shorten installation timelines and broaden eligibility sites. What is still less well-characterized, mainly because it is rarely reported outside vendor sources, are energy use under real clinical duty cycles, uptime and unplanned service rates, and the long-horizon reliability of cryocoolers. Independent metering and site-level audits should therefore accompany procurement decisions.

Uncertainties and Downsides

The current literature leaves several questions open. Long-term cryocooler reliability and service response times are not well-described. Artifact profiles and susceptibility behavior at lower fields vary with sequence design and coil options. Energy consumption reported in marketing materials may not reflect clinical workload. Moreover, in hot climate regions, heating, ventilation, and air conditioning (HVAC) demand may offset some of the energy advantages. Finally, application ecosystems (advanced coils and specialized sequences) are still maturing on some sealed-helium platforms.

Limitations of the Evidence and This Review

This review relied in part on vendor-reported materials, which may overestimate performance and should be interpreted with caution. Clinical studies are few, heterogeneous, and often small. Operational metrics are inconsistently reported and frequently vendor-supplied. We therefore did not attempt a meta-analysis and instead distinguished independent reports from manufacturer claims.

Future Directions

Multicenter, reader-blinded comparative studies anchored to 1.5 T and 3 T protocols are needed to test diagnostic equivalence across common indications. In parallel, a minimum operational dataset should include helium inventory (L), vent-pipe requirements, scanner mass (kg), installation time, energy (KWh/day), uptime (%), and unplanned service events. Transparent disclosure of vendor involvement and funding will strengthen credibility and facilitate synthesis. 

Clinical Protocol Considerations

Reader-blinded series at low and mid field consistently emphasize protocol tailoring. T2-weighted and proton density-weighted sequences can be adequate for musculoskeletal assessments when optimized for lower intrinsic signal-to-noise ratio (SNR), while diffusion-weighted and susceptibility-based imaging often require parameter adjustments to preserve lesion conspicuity. Head and joint imaging benefit from receive-coil proximity and parallel imaging with conservative acceleration factors. Across studies, the combination of careful shimming, longer repetition time (TR)/echo time (TE) windows where tolerable, and reconstruction refinements mitigates, but does not eliminate, the contrast and resolution gap relative to 1.5 T/3 T comparators.

Implementation and Service

Operational experience remains unevenly reported. Sites that deployed sealed-helium systems cite simplified siting (no quench pipe in some jurisdictions), shorter installation windows, and fewer cryogen-related service calls. Counterbalancing considerations include cryocooler maintenance schedules, the need for vigilant site HVAC control to ensure thermal stability, and the maturation of vendor-specific coil/sequences. Prospective logging of uptime, unplanned service, and energy use under defined duty cycles would materially improve cross-site comparability.

## Conclusions

Sealed-helium MRI is operationally promising and, with sustainable protocols, clinically usable for selected indications. Broader adoption should be paced by independent clinical comparison and standardized operational reporting to ensure siting gains translate into reliable, cost-aware care.
